# Ritualized relational autonomy in Chinese end-of-life care: balancing Confucian benevolence and individual rights

**DOI:** 10.3389/fpubh.2026.1811046

**Published:** 2026-08-10

**Authors:** Xun Ma, Zepin Lin

**Affiliations:** 1 School of Philosophy, Fudan University, Shanghai, China; 2 Institute of Technology Ethics for Human Future, Fudan University, Shanghai, China; 3 Department of Philosophy, Xiamen University, Xiamen, China

**Keywords:** bodily autonomy, end-of-life care, family mourning, ren’ai (benevolence), ritualized relational autonomy

## Abstract

This article develops a hypothesis-and-theory framework for ethical tensions in contemporary Chinese end-of-life care and family mourning. It examines the relationship between Confucian ren’ai (benevolence), expressed through embodied familial care, and modern rights-based commitments to bodily autonomy, dignity, and informed choice. The article offers a conceptual and normative analysis informed by interpretive cultural inquiry and existing empirical research on family-centered decision-making, advance care planning, caregiver burden, and hospice and palliative care in China. It argues that the body is the key site at which benevolence and rights intersect: in Confucian ethics, the body is the medium of care, devotion, and mourning, while in modern bioethics it is the bearer of autonomy and consent. On this basis, the article proposes a framework of ritualized relational autonomy, structured through three stages: care agreements, family meetings, and care-termination rituals. This framework aims to prevent benevolent care from hardening into unlimited liability while also avoiding an atomized model of autonomy detached from family life. The article further argues that this framework has practical significance for public health ethics in aging societies by clarifying how family care, patient dignity, caregiver support, and bereavement may be more effectively coordinated under contemporary Chinese conditions.

## 1 Introduction

In end-of-life care and post-bereavement mourning, contemporary Chinese families often find themselves pulled between two ethical orientations. On the one hand, there is the Confucian tradition of ren’ai (仁爱, benevolence), which emphasizes attending to, accompanying, and mourning one’s relatives through concrete bodily involvement. On the other hand, there is the modern rights-based consciousness of individual rights, which insists that even in situations of extreme vulnerability, each person retains rights to bodily autonomy, dignity, and informed choice. These two orientations frequently appear to conflict. For example, out of filial piety concern, a family may conceal a diagnosis or make decisions on behalf of the patient, thereby violating the medical-ethical requirement to respect autonomous choice. Such tensions reveal the difficulties of adapting traditional Confucian ethics to a modern and increasingly individualized social world. Yet they are not irreconcilable opposites. This article argues that the two perspectives can in fact be mutually complementary: benevolence embodies profound moral emotions and caregiving practices, while rights-based norms provide indispensable limits that prevent care from overstepping its bounds and infringing upon personal wishes.

The central question, then, is how contemporary family practice in mainland China can balance these orientations—how it can sustain the affective care characteristic of tradition, marked by bodily devotion, bedside tending, and accompaniment through the final moments of life, while also ensuring that individual bodily autonomy and dignity are not submerged by collective will. This question is of both theoretical and practical importance. It concerns not only the interpretation of Confucian ethics under modern conditions, but also broader issues in public health ethics, palliative care, caregiving burden, and end-of-life decision-making in aging societies. In contemporary China, these issues arise within a context in which family-centered care remains highly influential, while advance care planning, hospice and palliative care, and modern informed-consent norms are developing unevenly across institutions and regions.

This article develops a hypothesis-and-theory account of this problem. It does not report newly collected empirical data; rather, it offers a conceptual and normative framework, informed by interpretive cultural analysis and existing empirical research on family caregiving, end-of-life decision-making, and advance care planning in China. The central hypothesis is that the tension between Confucian benevolent care and modern individual rights can be more sustainably addressed when caregiving is not left either to implicit filial obligation or to abstract individualism alone, but is instead structured through explicit, relational, and culturally meaningful procedures. To capture this possibility, the article proposes a framework of ritualized relational autonomy.

The framework proceeds from the claim that the body is the crucial site at which these ethical orientations intersect. In Confucian ethics, the body is the medium through which familial affection, care, and mourning are enacted. In modern bioethics, the body is also the bearer of autonomy, informed consent, and dignity. Because the body stands at the intersection of these two normative registers, it provides an especially fruitful point of entry for understanding both the conflict and the possible reconciliation between them. The article therefore treats the body not merely as an object of care or grief, but as the practical and symbolic locus through which benevolence and rights can be brought into a more productive relation.

On this basis, the article advances the framework of ritualized relational autonomy as a way of balancing Confucian benevolent care with modern individual rights in contemporary Chinese end-of-life care and family mourning. More specifically, it argues that the ethical tensions surrounding diagnosis disclosure, medical decision-making, caregiver responsibility, and post-bereavement moral residue can be mitigated when caregiving is organized through a three-stage structure: bounded care agreements, deliberative family meetings, and care-termination rituals. The contribution of the article is therefore not to offer an empirical intervention study, but to formulate a theoretically grounded and culturally situated model that may guide future empirical research and practice design in end-of-life care.

The remainder of the article proceeds as follows. Section 2 examines the body as the site where care, mourning, benevolence, and autonomy intersect, and clarifies the ethical tension between Confucian embodied care and modern rights-based norms. Section 3 situates this tension within the empirical and public health context of contemporary China, with particular attention to family-centered decision-making, advance care planning, caregiver burden, and intergenerational as well as urban–rural differences. Section 4 develops the framework of ritualized relational autonomy through three stages—separation, liminality, and aggregation—corresponding, respectively, to care agreements, family meetings, and care-termination rituals. Section 5 addresses major philosophical objections while also elaborating the framework’s practical significance for public health ethics and end-of-life care. Section 6 concludes by summarizing the article’s theoretical contribution and indicating directions for future empirical research.

## 2 The body, benevolence, and autonomy in end-of-life care

The ethical tensions surrounding end-of-life care and family mourning in contemporary China are most clearly visible when viewed through the body. The body is not merely the object of medical intervention or the biological site of dying; it is also the medium through which care, grief, obligation, and dignity are expressed and contested. In contexts of serious illness, dying, and bereavement, bodily practices such as feeding, cleaning, turning, accompanying, washing, and commemorating become charged with moral meaning. For this reason, the body occupies a double normative position: in Confucian ethics, it is a site of familial affection and embodied benevolence; in modern bioethics, it is also the bearer of autonomy, informed consent, and individual dignity. The central tension explored in this article arises precisely because these two normative registers converge upon the same bodily reality.

In mourning, the body has a distinctive philosophical significance. On the one hand, the body of the deceased is the immediate bearer of the transition between life and death: family members often come to grasp the reality of death through attending to, gazing upon, and bidding farewell to the corpse. On the other hand, the bodies of the living remain active in mourning, since survivors establish moral and affective relations with the deceased and with one another through bodily presence and action. This is especially evident in Chinese cultural settings, where grief is frequently enacted through concrete bodily practices, forming what may be called an embodied mode of mourning. Rituals such as keeping vigil, observing mourning, holding the funeral bier, and conducting burial all depend upon bodily participation. In traditional Confucian funerary rites, relatives wash and dress the body, kneel and weep, and keep vigil without sleep, using bodily exertion to express reverence and sorrow (1, 2). Even in contemporary families, the care of a dying relative—bringing water and medicine, turning the body, wiping and cleaning—and bodily gestures at funerals, such as embracing, supporting, and accompanying one another, remain crucial ways of sustaining grief and care together. Through such practices, mourning becomes not merely an inner feeling but an enacted relation.

Confucian thought gives this embodied dimension of mourning and care an explicit ethical significance. Confucius emphasizes qin qin, or affection for one’s close kin, and holds that the grief arising from the death of one’s parents should be shaped and expressed through ritual (2). The Analects links the three-year mourning period to the dependence of the child upon parental care, thereby presenting grief not simply as spontaneous emotion but as morally structured response (2, 3). Xunzi develops a related point: grief, if left entirely unchecked, may become either deficient or excessive, and therefore requires ritual shaping so that it acquires fitting form and order (1). On this view, ritual has a dual function. It expresses genuine feeling, but it also disciplines feeling, preventing numbness on the one hand and destructive excess on the other. Embodied ritual thus mediates between inward sorrow and outward ethical order.

This emphasis on embodiment is not confined to mourning narrowly conceived. In end-of-life care, bodily action remains the principal vehicle through which familial concern is enacted. Within Confucian ethics, benevolence is not merely a psychological attitude but a practical and relational virtue. In *The Analects,* Confucius famously states that “the benevolent person loves others,” and this love begins with those to whom one stands in the closest familial relation (2). The *Classic of Filial Piety* further connects the body to filial obligation by stressing that one’s body is received from one’s parents and should therefore be treated as the beginning of filial piety (4). In family care for frail or dying parents, this moral logic takes the form of highly concrete bodily attentiveness: bringing soup and medicine, cleaning the body, assisting with toileting, remaining at the bedside, and accompanying the parent through the final stage of life. Such acts are not usually understood as optional kindnesses, but as morally weighty familial responsibilities.

At its strongest, this Confucian ethics of embodied care expresses the moral depth of ren’ai (benevolence). Bodily care is not external to affection; it is one of its principal languages. Feeding, washing, accompanying, and later preparing the body of the deceased all communicate love and respect in ways that cannot be reduced to abstract goodwill. In this sense, bodily care possesses irreducible ethical significance. It creates and sustains a form of intimacy in which caregiver and cared-for are bound together through shared vulnerability and practical attention. Zehou (5) describes the family as a blood-kinship community in which everyday life and mutual care generate a shared world of feeling and fate. Contemporary research similarly shows that, in Chinese cultural contexts, caring for one’s parents carries strong moral meaning and is often treated as central to being a decent person (6). Filial care is therefore not merely a private sentiment; it is also embedded in a broader ethical vision in which the family serves as the starting point for moral life and the cultivation of social virtue (7, 8).

At the same time, however, the ethical strength of embodied benevolence also reveals its principal danger. Because care is morally valorized and linked to filial duty, it can slide into what may be called unlimited liability: an expectation that children should absorb boundless caregiving burdens regardless of personal cost, competing obligations, or the wishes of the care recipient. In traditional and contemporary settings alike, caregiving is often presumed to demand sacrifice without clear boundaries, thereby turning a virtue of care into an open-ended moral imperative (9–13). In such situations, caregivers may experience prolonged physical exhaustion, resentment, and moral guilt, while still feeling unable to withdraw because withdrawal appears indistinguishable from unfilial conduct. Recent studies of caregiving in China likewise point to structural imbalance, heavy burden, and the unequal distribution of responsibility in family-based care for older adults, particularly under the demographic and social conditions facing the one-child generation (14–16). What begins as benevolence can therefore harden into a boundaryless expectation of self-sacrifice.

This danger becomes especially acute when embodied care intersects with modern norms of bodily autonomy and individual rights. Modern bioethics treats respect for autonomy as a core principle, emphasizing that each person has decision-making authority over their own body and life (17, 18). In end-of-life settings, this includes the right to know one’s diagnosis, the right to participate in decisions concerning treatment, and the right to refuse interventions that may prolong life at the expense of dignity or quality of life. From this perspective, care that overrides a person’s expressed wishes—even when motivated by concern—risks becoming paternalistic or coercive [Deutscher (19, 20)]. Rights-based ethics therefore insists that bodily vulnerability does not nullify personhood; rather, it is precisely in moments of dependency that dignity and consent become especially important.

Yet the introduction of autonomy into the Chinese family context often generates tension rather than immediate clarity. Traditionally, the family is regarded as a moral whole, and deference to elders or collective decision-making is often treated as virtuous. By contrast, modern bioethics requires that individuals be recognized as rights-bearing subjects whose wishes cannot simply be absorbed into familial authority (6). This conflict is not merely abstract. It appears in practical questions such as whether a terminal diagnosis should be disclosed directly to the patient, whether aggressive treatment should be continued, and whose judgment should prevail when physicians, patients, and relatives disagree. In many Chinese medical contexts, physicians still communicate first with family members and allow them to decide whether, how, and when the patient should be informed, often on the assumption that this protects the patient from distress (21–23). However, available evidence also suggests that many older adults in China wish to know the truth and to retain some role in end-of-life decision-making, rather than being wholly shielded by the family (6, 24). The real difficulty, then, is not whether autonomy matters in Chinese contexts, but how autonomy should be negotiated within deeply relational forms of life.

The conflict is further intensified by intergenerational and socio-cultural change. In many contemporary Chinese families, older generations remain attached to strong norms of filial obligation and family authority, whereas younger generations, shaped by modern education and rights discourse, place greater weight on personal wishes, informed consent, and quality of life. This can generate fracture within the family precisely at the moment when coordination is most needed (6, 14, 15, 25, 26). Similar tensions may also vary across social settings, including urban–rural differences and differences associated with educational background, although the implications of such variation require fuller empirical discussion in the following section. What matters here is that the clash between benevolence and autonomy is not simply a clash between “Chinese tradition” and “Western modernity,” but a conflict internal to contemporary Chinese family life itself.

Accordingly, neither of the two dominant moral orientations is sufficient on its own. If familial benevolence is left without boundaries, it may turn into intrusion, exhaustion, and moral coercion. If personal autonomy is detached from relations of dependence and care, it may become overly atomized and inattentive to the lived conditions of dying, grief, and family obligation. What is needed is not the triumph of one side over the other, but a mediating framework capable of preserving the moral warmth of embodied care while also protecting the individual’s status as a rights-bearing subject. The theoretical task, therefore, is to identify a model in which familial care remains meaningful, yet does not collapse into unlimited liability; and in which autonomy remains protected, yet is not imagined as if it were exercised outside all relationships. It is this task that motivates the framework of ritualized relational autonomy developed later in the article.

For present purposes, the conclusion of this section is straightforward. The body is the crucial site at which Confucian benevolence and modern autonomy come into both conflict and contact. It is through the body that familial affection becomes care, and through the body that dignity and consent become practically urgent. The problem of end-of-life care in contemporary China is therefore not simply whether one should choose benevolence or rights, but how bodily care, mourning, and decision-making can be normatively organized so that neither care nor autonomy destroys the other. The next section situates this problem within the empirical and public health context of contemporary China, thereby clarifying why such a mediating framework is not only conceptually desirable but practically necessary.

## 3 End-of-life care in contemporary China: empirical and public health context

The normative tension discussed in the previous section is not merely conceptual. In contemporary China, end-of-life care unfolds within a rapidly aging society, an expanding but still uneven palliative-care system, and a family-centered moral culture that continues to shape medical communication and decision-making. Recent overviews of healthy ageing and hospice development in China indicate both real policy progress and persistent structural gaps. On the one hand, China has expanded hospice and palliative-care services through pilot programs, standards development, training efforts, and public education; on the other hand, service provision remains uneven across regions and institutions, and the integration of palliative care into routine eldercare and community care is still incomplete (27). Policy analyses likewise describe the current phase of hospice development as one of accelerated institutionalization since the national pilot initiatives launched in 2017, with subsequent expansion to a much wider set of pilot cities and districts, yet still marked by uneven implementation and limited depth of service delivery (28). These conditions make end-of-life decision-making not only a family matter, but also a public-health issue concerning service capacity, care coordination, and support for older adults and their caregivers.

Within this broader setting, one of the most salient empirical features of end-of-life care in China is the continuing importance of family-centered decision-making. Existing studies suggest that, in many Chinese clinical contexts, serious diagnoses and prognostic information are still frequently filtered through family members before being disclosed to patients, and treatment decisions are often negotiated at the family level rather than treated as the exclusive prerogative of the individual patient (21–23). This pattern is neither accidental nor merely institutional inertia. It reflects a cultural logic in which family members understand themselves as morally responsible for protecting the patient from distress, preserving hope, and bearing the consequences of treatment decisions together. Yet the same literature also shows that this protective logic can conflict with patient preferences for truth-telling, self-determination, and advance planning. Woo (6) argues that the common assumption that “Chinese culture does not value autonomy” is too crude, while Yue et al. (24) show that Chinese older adults’ engagement with advance care planning is shaped less by a simple rejection of autonomy than by a complex process of readiness, relationship, and contextual trust. The empirical reality, therefore, is not a simple absence of autonomy, but an unstable negotiation between family protection and patient voice.

This point becomes especially clear in the growing literature on advance care planning (ACP) in Chinese contexts. Earlier work already showed that many older Chinese adults expressed interest in discussing end-of-life care preferences and healthcare autonomy, even if formal ACP awareness and uptake remained limited (48). More recent studies continue this picture of ambivalent openness. Cross-sectional and qualitative work suggests that acceptance of ACP is rising, but that actual engagement remains uneven and highly dependent on cultural framing, prior end-of-life experience, communication style, and the availability of supportive information (24). Research on informal ACP discussions among older Chinese adults further suggests that education level and media access matter: education and social-media use were associated with differences in ACP discussion patterns, which indicates that receptivity to planning is socially stratified rather than uniform across the older population (29). Other studies stress that ACP in Chinese settings is more likely to succeed when it is framed relationally and introduced through culturally sensitive tools rather than through purely legalistic or highly individualistic language (14, 15, 49). What emerges from this body of work is not simply a deficit model—China as “not yet ready” for ACP—but a more specific lesson: ACP requires cultural adaptation if it is to function within family-centered moral worlds.

The same need for cultural adaptation appears in institutional and community practice. Studies of Chinese community health workers, older adults in long-term care, and program-design efforts in mainland China and Chinese-speaking settings indicate that ACP and family-oriented end-of-life communication are feasible, but only when implementation accounts for local relational norms, workforce capacity, and communication barriers (30, 31). A recent randomized controlled trial among community-dwelling older adults with chronic diseases in China reported the feasibility and acceptability of a theory-based ACP intervention, while qualitative work in nursing homes and surrogate decision-making contexts similarly suggests that hospice and ACP-related discussions can be meaningful when they are embedded in trust, guided communication, and role-sensitive facilitation (32, 33). These findings are important for the present article because they suggest that the issue is not whether deliberative structures such as family meetings or negotiated planning are alien to Chinese practice. Rather, the problem is that such practices remain unevenly institutionalized, insufficiently routinized, and often disconnected from a broader ethical framework capable of integrating family care, patient dignity, and caregiver sustainability.

The question of caregiver burden further strengthens the public-health relevance of the problem. Chinese end-of-life care still relies heavily on family labor, especially in serious illness and advanced aging. Research on family caregivers in palliative contexts in China describes high levels of burden, unmet support needs, emotional strain, and vulnerability to compromised quality of life, particularly when daily care time is long, social support is weak, and care coordination is poor (34, 35). Qualitative studies in Beijing and elsewhere similarly show that family caregivers often struggle with uncertainty, competing obligations, practical exhaustion, and the need to improvise coping strategies across hospital and community settings (34). Recent work on medication management and bereavement experiences also indicates that family caregivers are expected to shoulder technically and emotionally demanding forms of care, often with inadequate institutional backing (36, 50). In other words, the danger of what this article calls “unlimited liability” is not only a philosophical diagnosis; it corresponds to empirically documented patterns of overload, asymmetrical responsibility, and moral stress among Chinese family caregivers.

These pressures are also distributed unevenly across social contexts. Existing evidence suggests important urban–rural, educational, and intergenerational differences in how eldercare obligations and end-of-life expectations are understood and managed. Liu et al. (14, 15), for example, show that the transition of eldercare responsibility and traditional filial-piety concepts in China exhibits significant urban–rural variation. More broadly, the literature on ACP discussion and engagement indicates that educational background, access to information, and communicative resources influence willingness and readiness to discuss end-of-life preferences (29, 37). At the family level, studies of intergenerational relations in contemporary China likewise suggest that younger and older generations often occupy different normative worlds: older generations may continue to treat deference, endurance, and family authority as morally central, whereas younger generations are more likely to invoke choice, quality of life, and self-determination (14, 15, 25, 26). These differences do not invalidate a common ethical framework, but they do show that any workable model must be flexible enough to operate across heterogeneous social settings rather than presupposing a single, homogeneous “Chinese culture.”

Taken together, the available empirical literature suggests a mixed assessment of current ethical practice in Chinese hospice and palliative care. Its strengths are considerable. Family participation remains strong; relational commitment often provides emotional continuity for seriously ill older adults; and recent policy and program development indicates growing institutional recognition of palliative and hospice needs (27). Yet the problems are equally clear. Patient voice can still be marginalized by protective family decision-making; ACP remains uneven in awareness and uptake; caregiver burden is often shouldered privately within families; and institutional mechanisms for structured family communication are still underdeveloped or inconsistently implemented (30–32, 34). The task, then, is not simply to import a Western autonomy model or to reassert an unbounded familial model, but to identify a practical ethical framework that can work under these empirical conditions. It is precisely here that the proposal of ritualized relational autonomy becomes relevant: it responds to a setting in which care is deeply relational, but where those relations require clearer procedures, negotiated boundaries, and stronger institutional support if they are to remain both humane and sustainable.

## 4 Ritualized relational autonomy: a three-stage framework

In light of the foregoing analysis, this article proposes ritualized relational autonomy as a normative framework for end-of-life care and family mourning in contemporary Chinese contexts. The framework seeks to preserve the moral warmth of Confucian benevolent care while also protecting bodily autonomy, informed consent, and the dignity of the person at the center of care. Its central claim is that the conflict between familial benevolence and individual rights is not best resolved either by leaving caregiving to tacit filial obligation or by treating autonomy as if it were exercised in isolation from family life. Rather, caregiving should be organized through explicit relational procedures that are ethically meaningful, practically workable, and culturally intelligible. In this sense, ritualization does not mean restoring traditional ritual in its historical form. It means creating structured practices through which care is bounded, decisions are negotiated, and responsibility is brought to a morally intelligible close.

This framework draws on van Gennep’s account of rites of passage, according to which major transitions are typically organized through three phases: separation, liminality, and aggregation. End-of-life care can be understood in these terms, not only as a transition in an individual life but also as a transition in a family’s ethical order. The three phases are therefore reformulated here as three practical mechanisms: a care agreement as a boundary-setting ritual of separation, family meetings as a liminal mechanism of negotiated autonomy, and a care-termination ritual as an aggregative practice of closure, acknowledgment, and release. Family-meeting guidelines in specialist palliative care already show that structured communication with families can be organized as a repeatable clinical-ethical process, while recent studies in Chinese community and long-term-care settings indicate that culturally adapted advance care planning and structured discussion are feasible when communication, context, and role expectations are taken seriously (30, 31, 38).

### 4.1 Separation: care agreement as a boundary-setting mechanism

In many Chinese families, the onset of serious illness activates a default moral script: close relatives are expected to step in immediately, absorb responsibility, and endure escalating demands in the name of filial care. The moral force of this expectation is understandable. Yet precisely because it is usually tacit, it often lacks clear limits, role allocation, or agreed procedures for revision. What begins as benevolence can therefore become a structure of silent escalation in which responsibility expands faster than deliberation. The purpose of the first stage is to interrupt that transition. A care agreement functions as a ritual of separation because it marks the passage from ordinary family life into a distinct period of intensified caregiving governed by explicitly discussed expectations rather than by unspoken moral absolutism.

The care agreement should not be understood narrowly as a legal contract. In the present framework, it is an ethical and relational document. Its point is not to juridify love, but to make responsibility speakable, distributable, revisable, and therefore sustainable. Research on ACP implementation in Chinese community and long-term-care settings suggests that structured discussion, contextual adaptation, and role clarification are central to workable end-of-life planning. Chinese community health workers have expressed willingness to implement ACP, but their willingness is shaped by attitudes, perceived norms, training, and practical support, while programme-theory work in Chinese long-term-care facilities likewise emphasizes context-sensitive mechanisms rather than abstract rights language alone (30, 31).

At minimum, the agreement should address five issues. First, it should record the patient’s preferences wherever these can be expressed, including diagnosis disclosure, preferred place of care, major treatment limits, and any prior wishes concerning resuscitation, tube feeding, or aggressive intervention. Second, it should allocate caregiving tasks across family members, specifying who will accompany hospital visits, who will provide daily care, who will manage finances, and when external help may be introduced. Third, it should identify limits: maximum care hours, respite periods, work constraints, or other boundaries beyond which continued care would become unsustainable. Fourth, it should specify how disagreement will be handled, for example by reconvening a family meeting, consulting clinicians, or deferring to a previously stated patient preference on core bodily matters. Fifth, it should include a review point, since serious illness is dynamic and any agreement that cannot be revised will soon become either irrelevant or oppressive. These clauses do not eliminate moral conflict, but they prevent conflict from remaining invisible until it erupts into resentment, guilt, or coercion.

The ethical significance of the care agreement lies in its boundary function. For caregivers, it creates a morally legitimate distinction between responsible devotion and self-erasure. For patients, it prevents benevolent care from becoming an unquestioned arrangement in which others decide, suffer, and sacrifice “for one’s own good” without their voice. For the family as a whole, it marks the beginning of caregiving as a shared undertaking rather than a diffuse emergency. In that sense, the agreement is ritualized because it solemnizes entry into a special period of moral labor. It also provides an important psychological cue: caregiving has an endpoint. That cue matters because one source of subsequent guilt is precisely the experience that one’s filial task was never clearly delimited and therefore can never be clearly completed.

### 4.2 Liminality: family meetings as a mechanism of negotiated autonomy

Once caregiving has begun, the family enters what van Gennep would call a liminal phase: an in-between period marked by instability, high emotional intensity, and the progressive transformation of roles. It is in this phase that both the moral power and the moral danger of family care are greatest. Because the family is gathered around a vulnerable body, it can become a site of profound solidarity; but because decision-making is urgent and emotionally saturated, the patient’s individual voice can also be submerged, and caregivers’ own limits can disappear beneath a collective sense of mission.

The practical mechanism proposed for this stage is the structured family meeting. The palliative-care literature provides a strong basis for such a mechanism. Hudson et al. (38) developed multidisciplinary clinical practice guidelines for family meetings in specialist palliative care, emphasizing preparation, clarification of purpose, appropriate participant selection, information sharing, emotional support, discussion of options, and follow-up review. This is important because it shows that family meetings are not merely aspirational moral ideals; they can be organized as a recognizably structured clinical-ethical practice. Recent Chinese studies on ACP and communication likewise suggest that structured family discussion is institutionally imaginable when communication is adapted to local relational norms and implementation conditions (30, 31, 38).

Within the present framework, a family meeting should have a simple but stable format. It should begin with a medical update, ideally delivered by a physician or nurse who can clarify the patient’s condition, likely trajectory, and available options. It should then include a patient voice segment, allowing the patient to express fears, preferences, treatment limits, or previously stated wishes whenever this is possible. If the patient lacks decision-making capacity, prior statements or family-supported interpretation should be explicitly identified as such rather than silently assumed. A third segment should be devoted to the family’s own account of concerns, burdens, and disagreements, since negotiated autonomy does not erase family interests but renders them discussable. A fourth segment should involve ethical clarification, whether by a clinician, social worker, or other facilitator, identifying where filial concern, treatment hope, fear of regret, and respect for autonomy are actually in tension. Finally, there should be a decision and review stage, where points of agreement are documented, unresolved disagreements are named, and a time for reassessment is set.

The point of this mechanism is not proceduralism for its own sake. It is to create a temporary communicative space in which family hierarchy can be softened without being denied, patient preference can be heard without pretending that the family is irrelevant, and care decisions can be made without collapsing into either paternal silence or atomistic choice. Negotiated autonomy is therefore not a model of isolated self-determination. It is a relational form of autonomy achieved through dialogue, mutual acknowledgment, and clarified responsibility. This is also the stage at which the introduction of external resources can be discussed most productively. In strong filial scripts, hiring professional caregivers or involving institutional services is often treated as morally suspect. Yet research on ACP implementation in Chinese settings suggests that the success of end-of-life planning depends heavily on support structures, communication channels, and realistic role expectations (30, 31). Under those conditions, professional support can be understood not as abandonment but as a negotiated extension of familial care.

### 4.3 Aggregation: care-termination ritual as a mechanism of closure

The third phase addresses a problem often neglected in both bioethics and family-care discussions: how caregiving ends. After the death of a loved one, many caregivers do not simply return to ordinary life. They often continue to experience themselves as burdened by unfinished duty, residual responsibility, or guilt over what might have been done differently. The role of a care-termination ritual is to respond to this moral residue. In van Gennep’s language, aggregation marks reincorporation into ordinary life after the liminal period has ended. In the present framework, the corresponding mechanism is a structured practice through which the family acknowledges that the period of emergency caregiving is over, affirms what was done, and symbolically releases participants from the role of caregiver.

This ritual need not be elaborate. In contemporary urban China, a modest and intentional format will often be more realistic than a highly formal one. What matters is not ceremonial grandeur but ethical function. A care-termination ritual should include at least four components. First, there should be a review of the caregiving period, naming the major decisions taken and the labor undertaken. Second, there should be mutual acknowledgment, in which family members thank one another and recognize that different forms of contribution—practical, financial, emotional, coordinative—were all part of care. Third, there should be an affirmation of the deceased person’s wishes and dignity, especially where final preferences about treatment, place of death, or funeral form were respected. Fourth, there should be a symbolic closure act: sealing or archiving the care agreement, reading aloud a final statement of completion, or explicitly declaring that the caregiving task has been fulfilled.

This final stage is not only psychologically useful; it is also morally important. Research on grief rituals indicates that structured symbolic action can shape bereavement trajectories, and recent longitudinal work suggests that the meaning attached to performed or prevented rituals matters for grief reactions over time (39). More broadly, meaning-making research in bereavement emphasizes the role of socially mediated interpretation in adaptation after loss (40). In the present framework, that general insight takes a more specific ethical form. Closure is not forgetting, nor is it severing attachment. It is the family’s acknowledgment that love need not prove itself through endless self-reproach. Where unbounded filiality can generate not only caregiving burden during illness but also endless guilt after death, a care-termination ritual creates a morally legible point at which responsibility can be released without dishonoring the deceased.

This third stage is also where the framework most clearly protects modern dignity without severing ties to family care. If the deceased had requested a simple funeral, or had refused certain treatments, then compliance with those wishes should itself be publicly recognized as an expression of respect rather than denounced as insufficient devotion. Relational care and autonomy converge here rather than compete. The family honors the deceased not by overriding the person’s wishes in the name of love, but by carrying them through in a form the family can acknowledge together.

Taken as a whole, the three-stage framework of ritualized relational autonomy is meant to organize the entire arc of end-of-life care, from diagnosis and role transition to decision-making under strain and, finally, to the moral completion of caregiving after death. A care agreement interrupts silent escalation; family meetings transform implicit conflict into structured negotiation; a care-termination ritual converts private burden into acknowledged closure. The framework does not presume that all families will adopt these mechanisms in exactly the same form, nor does it claim that they have already been empirically validated as interventions. Its claim is more modest: under contemporary Chinese conditions, where family care remains morally central but institutional support and procedural clarity are uneven, such mechanisms provide a plausible normative model for bringing benevolence and autonomy into a more sustainable relation.

## 5 Philosophical justification and practical significance

Several objections may be raised against the framework of ritualized relational autonomy. The most important concern is that, by emphasizing relational negotiation, the framework may dilute individual rights and allow family influence to override patient autonomy. This concern is serious, especially in end-of-life care, where vulnerability, asymmetrical dependency, and emotional pressure can easily distort decision-making. Yet the alternative of treating autonomy as if it were exercised in complete social isolation is equally inadequate. Recent work in medical ethics argues that relational autonomy is most defensible when it does not replace autonomy with familial preference, but instead clarifies the conditions under which patients’ competence, authenticity, and relationships can be held together in decision-making. On this view, relational autonomy is not a license for family domination; it is a framework for distinguishing supportive involvement from undue pressure (41, 42). In this article, the point of negotiation is therefore not to subordinate the patient to the family, but to ensure that autonomy is exercised under conditions that acknowledge dependency, communication, and the practical consequences of dying within a family setting. The patient’s core bodily interests—such as disclosure preferences, refusal of burdensome interventions, and final wishes concerning the manner and place of death—remain normatively central, while the family’s role is made visible and discussable rather than covertly decisive.

A second objection is that ritualization risks making family care cold, bureaucratic, or overly procedural. If care is organized through agreements, meetings, documentation, and closure practices, it may appear that familial love has been displaced by administration. This objection, however, rests on too sharp a contrast between affect and form. Rituals are not external additions to care; they are often among the principal ways in which care becomes recognizable, shareable, and endurable. In palliative settings, patients and relatives consistently report that effective communication involves not only information exchange, but also the creation of a safe, respectful, and emotionally intelligible space in which concerns and preferences can be voiced (43). Research on family participation in end-of-life communication likewise suggests that structured involvement can improve understanding, trust, and the quality of shared decision-making, even though it must be managed carefully to avoid coercion or misunderstanding (44). The framework proposed here should therefore not be interpreted as replacing love with paperwork. Its aim is to provide forms through which love does not collapse into confusion, silence, or limitless obligation. A signed care agreement, a properly convened family meeting, or a simple closure ritual can function not as cold procedures, but as morally legible acts through which family members acknowledge one another’s responsibilities and limits.

A third objection is that the framework may appear culturally unstable: too “Western” for Confucian ethics because it includes rights, contracts, and autonomy, yet too “familial” for liberal bioethics because it resists treating the individual as the sole locus of moral authority. This concern should be addressed directly. Contemporary Chinese end-of-life care already unfolds within a hybrid normative environment. It is shaped simultaneously by filial expectations, biomedical institutions, informed-consent norms, evolving hospice policy, and public-health responses to population ageing. Recent reviews of hospice and palliative-care development in China describe exactly this kind of mixed environment: policy expansion and institutional experimentation have advanced quickly in recent years, but implementation remains uneven, and family-centered moral practices continue to structure much of what happens at the bedside and in community settings (27, 28). Under these conditions, insisting on a purified return either to premodern filial norms or to a fully atomized autonomy model is less realistic than identifying a principled framework capable of mediating between already coexisting moral logics. Ritualized relational autonomy is offered as such a mediating framework. It does not treat Confucian benevolence and modern rights as naturally harmonious, but neither does it treat them as mutually exclusive. Instead, it argues that family care can remain morally significant only if it acquires clearer limits, and that rights can remain practically meaningful only if they are articulated through the relationships in which dying actually occurs.

A fourth concern is more pragmatic: even if the framework is normatively attractive, it may be too idealized for real clinical settings. Families under stress may not want formal meetings; clinicians may lack time; community services may be thin; and some patients may be too ill or too reluctant to participate in explicit planning. This objection is well taken. The framework should not be presented as if it were a universally applicable protocol that can be imposed on all families in the same way. It is better understood as a structured ethical model whose components can be adapted in degree and form. Existing research on palliative communication, family meetings, and caregiver engagement supports this modest understanding. Clinical guidelines for family meetings in palliative care already show that such meetings can be scaled to context and adjusted to clinical realities (38). Broader evidence on structured communication with family caregivers indicates that even relatively modest procedural supports can improve safety, role clarity, and care coordination (45). Chinese studies on ACP implementation further suggest that success depends not on abstract commitment alone, but on contextual fit, communication skills, role clarity, and institutional support (30, 31). The practical significance of the present framework lies precisely in its intermediate status: it is more structured than a purely aspirational appeal to “communicate better,” but less rigid than a one-size-fits-all policy mandate.

The practical significance of this framework is especially visible when it is situated within public health. In ageing societies, end-of-life care is never only a private moral issue; it is also a question of service organization, caregiver support, institutional capacity, and inequity across regions and care settings. Recent analyses of hospice and palliative care in China emphasize both the expansion of services and the persistence of disparities in access, infrastructure, and implementation depth, especially across community, primary-care, and long-term-care settings (27, 51). In that context, ritualized relational autonomy offers more than a philosophical compromise. It provides a way of linking ethical clarity to practical organization. Care agreements can help distribute caregiving labor and reduce silent overburdening; family meetings can improve communication and reduce avoidable conflict around goals of care; care-termination rituals can support bereavement adjustment and reduce moral residue among relatives. None of these mechanisms eliminates structural shortages, but all of them help translate broad values—benevolence, dignity, solidarity, autonomy—into repeatable practices that institutions can support rather than merely presuppose.

The framework also has significance for caregiver burden, which has become increasingly visible in both palliative-care research and public-health discussion. Family caregivers in palliative settings often experience substantial physical, emotional, and logistical strain, and studies increasingly show that communication quality, role clarity, and support structures are significant factors in whether care becomes sustainable or overwhelming (34, 43). More recent intervention research suggests that family-centered or dyadic approaches may help reduce anxiety, burden, and distress among caregivers, even though the quality and settings of the evidence vary (14, 15). From this perspective, the framework’s insistence on negotiated boundaries is not a departure from benevolence but one of the conditions under which benevolence can remain durable rather than self-destructive. A family that explicitly discusses limits, roles, and review points is not thereby less loving; it may in fact be more capable of sustaining care without transforming devotion into resentment or collapse.

The framework further bears on the design of culturally appropriate end-of-life services. Recent qualitative work on home end-of-life care in China identifies communication strategies, practical support, stable care relationships, and the realization of end-of-life preferences as key elements of quality care (46). This is strikingly consistent with the concerns developed in the present article. A good death in such settings depends not only on symptom management or treatment withdrawal decisions, but on whether patients, families, and professionals can build enough trust to articulate and carry through what matters most. Ritualized relational autonomy supplies a conceptual structure for that task. It frames quality care not only as effective treatment or compassionate presence, but as the successful coordination of embodied care, relational trust, and respect for the person’s wishes. In this way, it can contribute to the ethical vocabulary through which Chinese hospice, home-based end-of-life care, and community palliative services are designed and evaluated.

Finally, the framework has significance for bereavement. End-of-life ethics often focuses on what happens before death—diagnosis disclosure, treatment choices, surrogate decisions—but the aftermath of care also matters. Research on meaning reconstruction in bereavement has shown that adaptation to loss is shaped by how survivors interpret what happened, what responsibilities were fulfilled, and what remains unresolved (47). Studies of grief rituals likewise indicate that the performance, disruption, or absence of meaningful ritual forms can affect mourning trajectories over time (39). The care-termination ritual proposed here responds to this dimension directly. Its purpose is not to close grief prematurely or to prescribe a single form of mourning, but to create a shared acknowledgment that caregiving has reached moral completion. In families where guilt can be prolonged by strong filial expectations and counterfactual self-reproach, this form of closure may be ethically as important as any earlier treatment discussion. It allows the living to continue their bond with the deceased without remaining indefinitely trapped in the idea that care can only prove itself through endless regret.

For these reasons, ritualized relational autonomy is best understood neither as a mere philosophical reconciliation of traditions nor as a finished clinical programme. It is a normative framework with practical significance because it clarifies what family-centered end-of-life care requires if it is to be both humane and sustainable under contemporary Chinese conditions. It rejects the idea that benevolence should remain undefined until it becomes burden, and it rejects the idea that autonomy is most authentic when stripped of all relational context. By organizing care through boundary-setting, deliberative communication, and morally intelligible closure, it offers a way of thinking about end-of-life care in which familial devotion and personal dignity need not be treated as adversaries, but can instead be coordinated within a more resilient ethical order.

## 6 Conclusion

This article has examined the ethical significance of the body in Chinese end-of-life care and family mourning, focusing on the tension—and the possibility of reconciliation—between Confucian benevolent care and modern ideals of bodily autonomy. The argument has been that these two ethical orientations, though often treated as oppositional, are not inherently incompatible. The body is the site at which they most visibly converge: in Confucian ethics, it is the medium through which familial affection, care, and mourning are enacted; in modern bioethics, it is the bearer of dignity, informed consent, and self-determination. For that reason, the problem is not whether one must choose benevolence or rights, but how bodily care, decision-making, and mourning can be normatively organized so that neither destroys the other.

On that basis, the article has proposed ritualized relational autonomy as a mediating framework. Drawing on the structure of rites of passage, it has reformulated end-of-life care as a three-stage ethical process. In the phase of separation, a care agreement makes responsibilities, limits, and patient preferences explicit, thereby interrupting the silent escalation of filial duty into unlimited liability. In the liminal phase, structured family meetings create a communicative space in which patient wishes, family concerns, and clinical realities can be negotiated without reducing autonomy to isolation or care to paternalism. In the aggregative phase, a care-termination ritual helps acknowledge completed responsibilities, honor the deceased’s wishes, and release survivors from endless guilt. Taken together, these mechanisms show that ritual and autonomy need not be enemies: ritual can give care durable form, while autonomy can be realized within relationships rather than outside them.

The broader significance of this framework lies in its relevance to contemporary Chinese public health. China has expanded hospice and palliative-care development considerably in recent years, yet the literature also emphasizes uneven implementation, regional disparities, and the continuing importance of family-centered care in actual end-of-life practice. Under these conditions, a workable ethical model cannot simply assume either the sufficiency of traditional familial obligation or the easy transplantation of an individualistic autonomy model. It must address caregiving burden, institutional constraints, communication gaps, and the need for culturally adapted planning in aging societies. Recent work on Chinese long-term-care facilities and older adults’ engagement with advance care planning suggests precisely this need for context-sensitive, relationally informed approaches.

The framework developed here does not claim to be a validated intervention. Its status is theoretical and normative. Its contribution is to clarify the form that a more humane and sustainable family-centered end-of-life ethics might take under contemporary Chinese conditions. Future empirical research should therefore examine whether the mechanisms proposed here—care agreements, family meetings, and care-termination rituals—can reduce caregiver burden, improve communication, increase meaningful patient participation, and support healthier bereavement trajectories across different institutional and social settings. Research on meaning reconstruction in bereavement further suggests that how survivors interpret care, loss, and unresolved responsibility remains central to adaptation after death (47), which strengthens the case for treating post-care closure as an ethical as well as psychological task.

In this sense, ritualized relational autonomy is best understood as a framework of mutual completion. It seeks to protect the dignity of the dying without isolating them from family care, and to preserve the moral depth of familial devotion without allowing it to become limitless sacrifice. When Chinese families confront the final transition between life and death, what is needed is neither a return to unbounded filialism nor the imposition of abstract individualism, but a more resilient ethical order in which care, dignity, and mourning can be held together.

## Data Availability

The original contributions presented in the study are included in the article/supplementary material, further inquiries can be directed to the corresponding author.
